# A non-local multi-head spatiotemporal attention LSTM for vehicle trajectory prediction

**DOI:** 10.1371/journal.pone.0357735

**Published:** 2026-09-21

**Authors:** Sidra Rashid, Muazzam A. Khan, Usman Akram, Raheel Mumtaz, It Ee Lee, Toqeer Ali Syed

**Affiliations:** 1 Department of Software Engineering, Bahria University, Islamabad, Pakistan; 2 ICESCO Chair Big Data Analytics and Edge Computing, Quaid-i-Azam University, Islamabad, Pakistan; 3 Department of Computer Sciences, Quaid-i-Azam University, Islamabad, Pakistan; 4 Department of Computer and Software Engineering, NUST CE & ME, Islamabad, Pakistan; 5 Faculty of Artificial Intelligence and Engineering, Multimedia University, Cyberjaya, Malaysia; 6 Faculty of Computer and Information System, Islamic University of Madinah, Madinah, Saudi Arabia; Southwest Jiaotong University, CHINA

## Abstract

Vehicle trajectory prediction (VTP) is the primary part of the perception-planning-control pipeline in autonomous driving. The inter-vehicle interactions strongly shape the trajectory of vehicles in complex traffic scenarios. Neural networks that use recurrent or convolutional architectures often exhibit performance degradation in longer prediction horizons. Many existing approaches confine interaction modeling to a predefined spatial neighborhood and a narrow temporal window, while implicitly assuming that all neighboring vehicles have equal influence on the future trajectory of target vehicle. This results in error accumulation and the inability to capture long-range spatiotemporal dependencies. To overcome these limitations, a non-local multi-head spatiotemporal attention based long short-term memory model (NL-MHA-LSTM) is introduced which employs an attention mechanism to assign context weights to relevant neighbor vehicles. It extends beyond pairwise effects to model long-range dependencies. The model emphasizes the most influential vehicles and formulate position-aware interaction representations. A comprehensive set of experiments is conducted on the pubicly available HighD dataset. The results demonstrate that the proposed model outperforms all state-of-the-art methods, achieving a 53.4% reduction in RMSE at the 5s prediction horizon relative to the next-best model. In addition, a detailed ablation study is conducted to systematically evaluate the impact of different attention mechanisms and varying numbers of attention heads on prediction accuracy across multiple horizons.

## Introduction

Accurate modeling of the surrounding vehicle ensures safe navigation and collision avoidance in autonomous driving. In real-world scenarios, when vehicle-to-vehicle (V2V) communication is not available, autonomous systems infer the intentions of surrounding vehicles using their on-board sensory data. A detailed analysis of vehicle interactions and historical trajectories is required to improve situational awareness [1]. Intelligent Transportation Systems (ITS) integrates data analytics, communication, and control in vehicular networks. Using real-time data from sensors and connected platforms, ITS improves mobility while addressing key challenges such as traffic congestion and accidents [2]. Autonomous vehicles follow a tightly integrated pipeline; perception, prediction, planning, and control [3]. Multi-modal sensors provide information about the environment, which helps to detect lanes, vehicles, and other road agents in real time. The limitations of individual sensors are overcome by a combination of sensor modalities for low-light and adverse weather conditions [4,5].

Traditional trajectory prediction methods are lightweight, but often struggle to model multi-agent interactions of dynamic traffic scenarios [6]. The shift toward data-driven methods has led to the adoption of deep neural networks, which often consider trajectory prediction as a sequence prediction task. In this framework, convolutional neural networks (CNN) and recurrent neural networks (RNNs) [7] have been extensively applied either individually or in combination with their advanced variants, namely gated recurrent units (GRUs) [8] and LSTM [9] networks. These architectures do not use any rules to capture inter-vehicle interactions and lane-changing behaviors.

Classical pooling schemes are computationally efficient, but often oversimplify the complex interactions, which decrease the overall prediction accuracy. Whereas, attention-based methods concentrate on the important dependencies and events in the sequence [10]. The recent literature has shown the high potentials of attention mechanisms for trajectory prediction [11].

In this paper, we propose a deep learning architecture based on LSTM with a non-local multi-head spatiotemporal attention mechanism (NL-MHA-LSTM) for vehicle trajectory prediction. Our model selectively aggregates the states of the most relevant surrounding vehicles regardless of their distance from the target vehicle. It also captures long-range critical dependencies in complex traffic scenarios. The model avoids any limitation of fixed spatial neighborhood while maintaining computational efficiency. A series of extensive experiments validate that proposed model works better than state-of-the-art methods on long prediction horizons in complex driving scenarios.

Vehicle trajectory prediction has become very popular in the last decade due to its significance in autonomous driving. A number of research works addressed this challenge, coming up with different modeling approaches and architecture designs. In this section, we narrow our research focus that specifically explores attention-enhanced LSTM architectures and their variants, highlighting how these approaches advance the state of the art in capturing complex spatiotemporal dependencies.

## 1. Related research

Social interactions have been considered for trajectory prediction in recent studies. Recurrent architectures, particularly LSTM networks, remain a cornerstone in capturing temporal dependencies [12]. LSTM-based approaches make use of the historical trajectory patterns to predict trajectories in extended horizons with a lower computational cost [13]. LSTM frameworks often integrate lane orientation and driving intentions to improve predictive performance [14]. Other studies improved LSTMs through multi-layer or hierarchical designs.

The ST-LSTM is proposed by Dai et al. [15]. It is a multi-layer model with two layers; the first layer learns the trajectory of an individual vehicle while the second one learns the interaction among the agents. Yang et al. [16] used similarity metrics on historical trajectories to improve prediction accuracy. The real time prediction of vehicle trajectory is also addressed using efficient LSTM-based architectures in dynamic traffic scenarios [17].

More advanced frameworks include the use of dedicated components or the combination of LSTMs with other models. Karimzadeh et al. [18] combined transfer and reinforcement learning to model urban traffic dynamics. Tang et al. [19] introduced stacked spatio-temporal layers along with an LSTM trajectory generator for modeling the motion of multiple agents. Chen et al. [20] designed a dynamic feature extraction component for capturing detailed interactions between vehicles. Min [21] proposed an architecture with hierarchical LSTMs to model driving intents and lane changes together with future trajectories.

Attention mechanisms address this challenge by adaptively focusing on the most informative components of the input, thereby enhancing the model’s capacity to identify critical dependencies and positional patterns within sequential data [10]. Their effectiveness in VTP has been demonstrated in recent works [11,22]. Attention mechanisms have been widely used in combination with LSTM and its variants. Messaoud et al. [23] designed an architecture based on LSTM which measures the effect of surrounding vehicles through an attention mechanism. Zhiming et al. [24] captured long-range dependencies to address the bottleneck of fixed-length context vectors in LSTM models. Jin et al. [25] designed a temporal attention mechanism within the LSTM decoder to identify events from historical trajectories. Building on this, Wang et al. [26] applied multi-head temporal attention to model vehicle behavior across different time scales, extracting rich interaction features through social tensors. Gao et al. [27] employed a similar approach of self-attention to capture global dependencies through scaled dot-product attention, generating context-aware trajectory representations. Qiao et al. [28] also applied self-attention to hidden LSTM states deriving attention-based adjacency matrices to reveal strong inter-vehicle dependencies.

**Fig 1:**
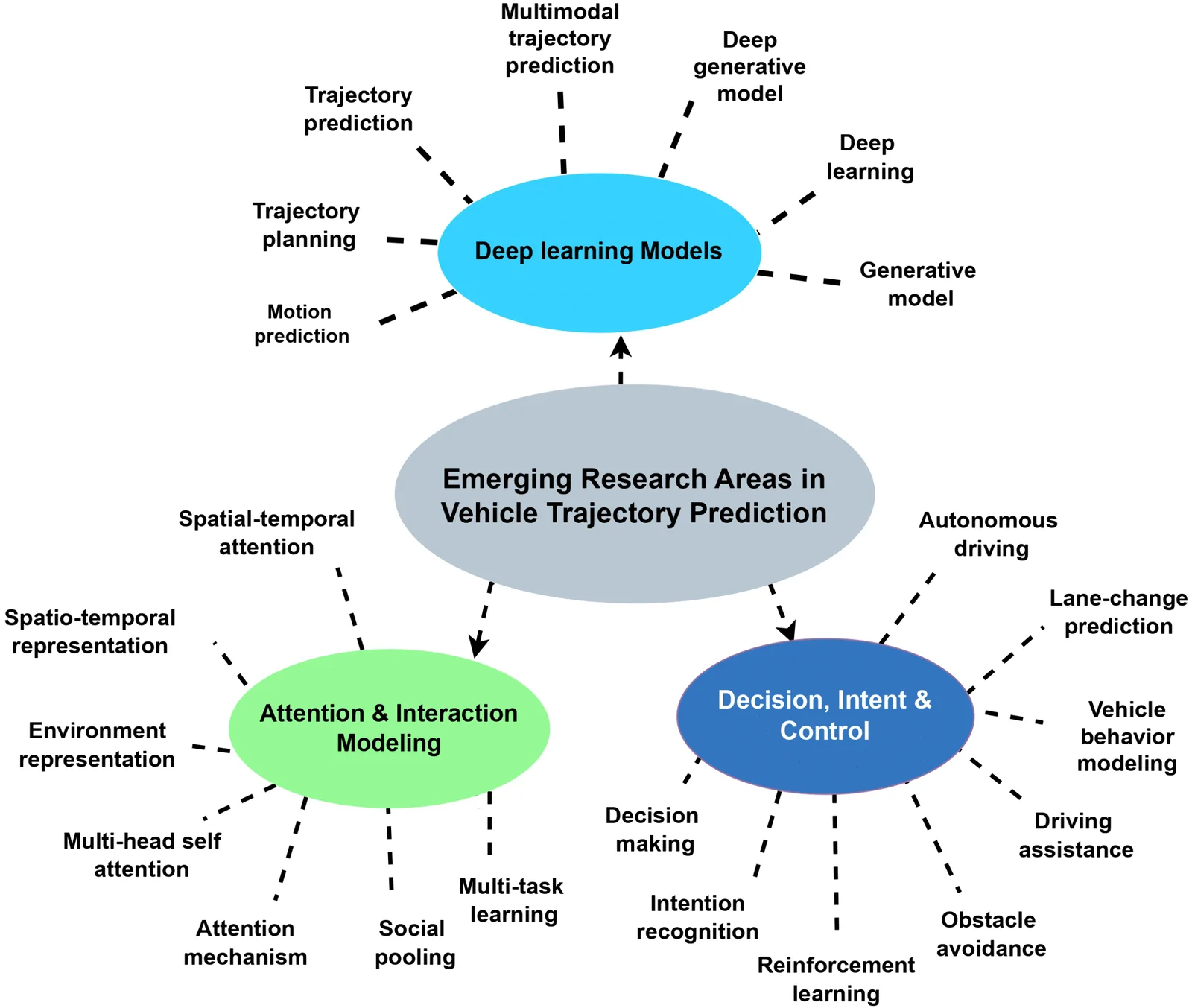
Emerging research areas in vehicle trajectory prediction.

In addition to LSTM-based models, attention mechanisms have been widely used with other deep learning architectures for trajectory prediction and related tasks. Cai et al. [29] utilized the graph attention network with convolutional social pooling in order to enhance trajectory prediction using contextual features. Similarly, Yuhuan et al. [30] introduced a temporal attention mechanism that adaptively weights scene images captured at different time intervals, inspired by channel attention in convolutional networks. This design computes pairwise attention scores between frames, allowing the model to focus on the most informative time steps. Tian et al. [9] incorporated attention with an encoder-decoder architecture based on interaction-based modeling for better trajectory predictions. Several other studies have incorporated transformer-based layers to encode agent-to-agent interactions in dynamic traffic scenarios [31–36]. Mo et al. [37] introduced edge-enhanced attention to predict trajectories for heterogeneous agents in complex traffic scenarios. The local graph and the temporal attention are combined to identify road structures [38]. An extension to this work introduces multi-head social attention layer. This layer enables models to selectively attend to spatially relevant neighbors while preserving long-range interaction modeling [39]. Spatiotemporal dual attention is employed to refine multimodal predictions by capturing dependencies in both historical and predicted future trajectories [40]. Cheng et al. [41] combined temporal attention for target vehicle history with spatial attention for surrounding agents. A hybrid framework is proposed that uses a graph attention network encoder for spatial interaction modeling and a transformer encoder for temporal dependencies [42]. Luo et al. [43] enhanced spatial attention by assigning adaptive weights to neighbors within a graph attention network (GAT). The diverse interaction patterns are captured using multi-head aggregation. Li et al. [44] integrated attention directly into the adjacency matrix allowing time-varying weights for connected nodes. A spatiotemporal attention model is developed that reflects human driving behavior, adaptively weighting both historical trajectories and influential spatial neighbors [45]. Similarly, Wang et al. [35] proposed an attention-based fusion framework that integrates multi-head self-attention to capture long-range temporal dependencies along with cross-attention mechanisms that capture interactions between traffic agents and the surrounding road infrastructure. A novel Lane-Specific Spatial-Temporal Attention Network (LSTAN) is introduced to handle the lane-level traffic data in predicting trajectories of vehicles [46]. More specifically, an encoder based on the long short-term memory networks is employed for learning the temporal information from both the target vehicles and the nearby vehicles. Lane attention module (LAM) and temporal self-attention module (TSAM) are designed for collecting the spatial and temporal information respectively. A trajectory prediction approach is proposed for highway weaving areas that considers the transfer properties of vehicle interactions as an integral part of the modeling process [47]. In particular, a hierarchical dynamic graph is used to consider transfer properties in two degrees of coupling; feature level and decision level. Thus, the problem of vehicle interactions modeling in mixed traffic is solved. Baicang et al. [48] proposed a novel multi-modal, end-to-end model for autonomous driving to predict vehicle speed and steering angle with high precision. The architecture utilizes spatial encoding, temporal dependencies and attention mechanism to identify critical features. Jin et al. [25] introduced STA-LSTM, a multi-modal trajectory prediction model to handle complex vehicle interactions in connected vehicle environments. The framework utilizes spatial grid occupancy to model interactions and employs convolutional pooling to extract dynamic interactive features. It effectively recognizes driving intentions and provides more reliable long-term trajectory predictions. Fig 1 provides insight into emerging research areas in vehicle trajectory prediction based on our literature survey.

Despite the fact that attention modules have proved to be effective at capturing the long-range temporal dependencies between relevant neighbors, lack of adequate analysis with respect to positional relevance and interactions dynamics may result in insufficient analysis of the traffic scene. Inaccurate choice of the attention heads may make the model miss important interactions, leading to low prediction accuracy. Our latest research presents an optimized LSTM architecture that utilizes spatio-temporal interactions to capture long-range dependencies without limiting interactions to any predefined spatial or temporal neighborhood [49].

In extension to our previous work, a non-Local multi-head spatiotemporal attention LSTM (NL-MHA-LSTM) is proposed for vehicle trajectory prediction. The proposed model captures a comprehensive range of interactions by selectively attending to the most relevant neighboring vehicles, without being constrained by all neighboring agents or predefined distance thresholds. Long-range dependencies can be efficiently modeled by it without adding any more trainable parameters into it.

This paper offers several distinctive contributions, including the following:

1. This work is an extension of our previous work [49]. A Non-Local Multi-Head Spatiotemporal Attention LSTM (NL-MHA-LSTM) is proposed for vehicle trajectory prediction. A multi-layer LSTM network is used for encoding trajectory history and relations among vehicles. The relevant neighbors are assigned attention weights using an attention mechanism. The LSTM decoder predicts trajectories by capturing long range dependencies.
2. An ablation study is performed to compare the performance of different attention mechanisms, attention heads and efficiency metrics.
3. The NL-MHA-LSTM model is evaluated on widely used HighD dataset, a well-established benchmark for highway vehicle trajectory prediction. Experimental results demonstrate that the proposed model achieves competitive performance relative to existing state-of-the-art approaches across long-range prediction horizons.

The structure of the paper is organized as follows. Section I provides an outline of the challenges and importance of vehicle trajectory prediction. Section II presents a literature review. Section III describes the architectural design of the NL-MHA-LSTM model. Section IV provides the experimental set up which includes implementation details, performance evaluation, models used for comparison and ablation study. Section V discusses experimental results, performance analysis, and comparative analysis of the proposed model. Finally, section VI concludes the paper and suggests directions for further research.

## 2. Overview of the proposed NL-MHA-LSTM model

**Fig 2:**
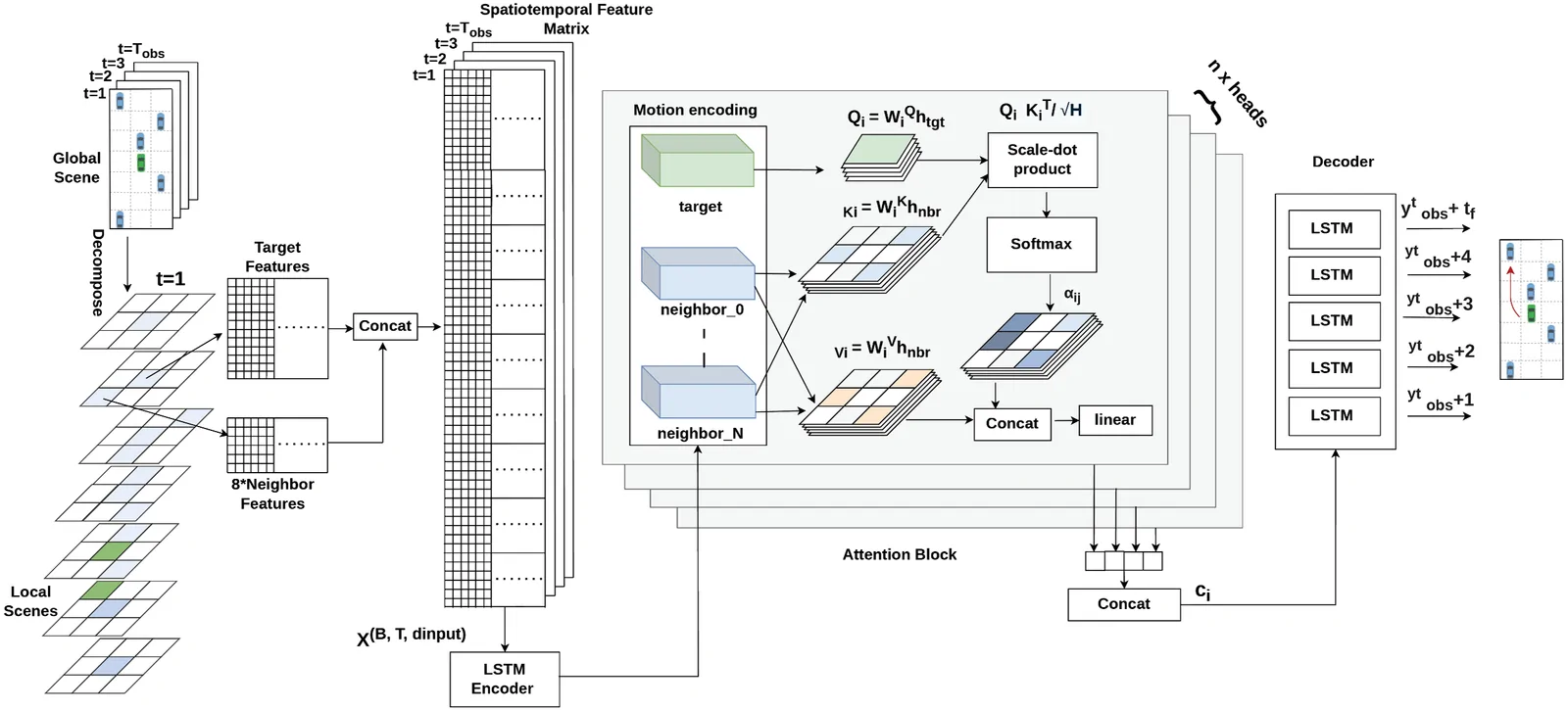
The proposed NL-MHA-LSTM model for vehicle trajectory prediction.

This section presents the architecture of the proposed NL-MHA-LSTM model. Fig 2 presents the proposed NL-MHA-LSTM. First, the global scene is decomposed into multiple time steps. These concatenated vectors are fed to the LSTM Encoder to learn temporal dynamics and interactions. A multihead attention block computes head-specific projections with similarity scores and a softmax over neighbors produces interaction weights. The contexts from all heads are then concatenated and passed through a linear layer to form a social embedding. The decoder then generates future positions for each time step. The design prioritizes behaviorally relevant neighbors without fixed distance thresholds and captures long-range dependencies efficiently.

The aim is to use the history of target vehicle and its interaction with neighbors to predict the future trajectory. Let $X_{t}=\{x_{t}^{0},x_{t}^{1},...,x_{t}^{N}\}$ represent the collection of all states of vehicles, where $x_{t}^{0}$ is target vehicle and $x_{t}^{i}$ is the neighbor vehicle for i≥1. In highway scenes modeled after HighD geometry (six lanes, three per direction), we adopt N≤8 as a practical upper bound corresponding to the usual interaction set: preceding, following, left-alongside, right-alongside, left-preceding, left-following, right-preceding, and right-following. This cap can be relaxed via adaptive neighbor selection that learns relevance scores per agent, or replace it with a fixed encoder for a graph-based architecture to scale to variable agents. The state vector $x_{t}^{i}$ could contain the planar position (*x*, *y*) along with many other features like velocity and acceleration. The superscript refers to vehicle id and the subscript shows the time step. The future positions of the target vehicle from *T* + 1 to *T* + *P* can be expressed as Eq 1.


$$\begin{array}{c} Y=\{y_{T+1}^{0},...,y_{T+P}^{0}\},\;y_{T+p}^{0}=({\hat{x}}_{T+p}^{0},{\hat{y}}_{T+p}^{0})\in {\mathbb{R}}^{2} \end{array}$$ (1)

Throughout this work, *P* refers to the prediction horizon, which spans from 1 to 5 seconds. All coordinates are in target-centric frame with origin at the target’s position at time *T*.

### 2.1 Non-local spatio-temporal feature extraction

The model input is defined as $X=\{X_{1},X_{2},...,X_{K}\}$ for all *K* tracks, where 1≤K≤60 reflecting the 60 tracks in the dataset. For this input, features of target vehicle and its neighbors are combined into a structured feature matrix. Each track has a different number of unique vehicles, we process every track and build a 60-dimensional feature vector for each vehicle at all timestamps during which it appears as a neighbor to another vehicle.

The term “non-local” means that all surrounding vehicles states are incorporated into the feature matrix globally, without imposing any spatial distance threshold or temporal window restriction throughout the observation window. Unlike local methods that restrict neighborhood to a fixed spatial boundary (10-15m) or temporal window (1-3s), our non-local formulation incorporates the states of all surrounding vehicles across the entire observation window without any proximity constraint. This is significant in lane-changing scenarios, where a distant vehicle in an adjacent lane may have a greater influence on the target vehicle’s future trajectory than a spatially closer one. This design guarantees that attention mechanism can access distant spatiotemporal interactions, which captures complete context to assign influence weights to surrounding vehicles, a richer foundation as compared to existing approaches. For the target vehicle, twelve features are extracted as detailed in Table 1, while six features are extracted for neighbor vehicle covering position (*x*, *y*), velocity $\left(v_{x},v_{y}\right)$, and acceleration $\left(a_{x},a_{y}\right)$. These are brought together into a unified non-local spatiotemporal feature matrix that serves as the input to the attention module. An insightful description of this matrix is provided in our previous work [49].

**Table 1:** Feature representation for target and neighboring vehicles.

Feature	Notation
Position	(*x*, *y*)
Velocity	$\left(v_{x},v_{y}\right)$
Acceleration	$\left(a_{x},a_{y}\right)$
Front sight distance	FSD
Back sight distance	BSD
Time-to-collision	TTC
Distance headway	DHW
Time headway	THW
Preceding vehicle velocity	$v_{p}$

### 2.2 LSTM architecture

The proposed NL-MHA-LSTM adopts a two-layer LSTM encoder-decoder architecture augmented with a multi-head attention mechanism that quantifies the influence of surrounding vehicles on the target vehicle’s future trajectory. The architecture consists of three core components: an encoder that processes the observed trajectory history, an attention module that learns to selectively weight inter-vehicle interactions, and a decoder that generates the predicted future trajectory based on the encoded context and attention-weighted representations. The mathematical equations that describe their functioning are given below.

#### 2.2.1 Encoder.

The encoder takes input sequences $𝐗=\{{𝐱}_{1},{𝐱}_{2},...,{𝐱}_{T}\}\in {\mathbb{R}}^{B\times T\times d_{\text{input}}}$, where *B* is the batch size, *T* is the sequence length, and *d*_input_ represents feature dimension. It iteratively updates its states as Eq (2) and outputs the terminal hidden and cell states $\left({𝐡}_{T},{𝐜}_{T}\right)$ that summarize the entire input sequence as Eq (3).


$$\begin{array}{c} h_{t},c_{t}=LSTM(x_{t},h_{t-1},c_{t-1}),\;t=1,...,T \end{array}$$ (2)

$$\begin{array}{c} \left(h_{t},c_{t}{)}_{t=1}^{T}⟶(h_{T},c_{T}\right) \end{array}$$ (3)

#### 2.2.2 Multi-head attention module.

After the LSTM encodes every vehicle’s motion history into a single vector, we feed the hidden terminal states into the attention module. In self-attention mechanism, each token or timestep forms a context-aware representation by attending to all others including itself. It considers a single attention head. This operation captures long-range dependencies in a translation invariant way and has become a standard mechanism for sequence modeling. To increase expressiveness and stabilize training, multi-head attention runs *H* independent attention heads in parallel, each with its own projections. The vehicle interactions are computed as follows:


$$\begin{array}{ccc} Q_{l} & = & W_{l}^{Q}h_{\text{tgt}} \end{array}$$ (4)

$$\begin{array}{ccc} K_{l} & = & W_{l}^{K}h_{\text{nbr},j} \end{array}$$ (5)

$$\begin{array}{ccc} V_{l} & = & W_{l}^{V}h_{\text{nbr},j} \end{array}$$ (6)

$W_{l}^{Q},W_{l}^{K},W_{l}^{V}$ are the learnable projection matrices in each attention head *l*. Each head thus captures a dynamics-rich representation of how every neighbour vehicle has been moving re*l*ative to the target vehicle. The *l*-th head for the target *i* aggregates neighbors via a softmax-weighted sum as Eq (7). Here, $\alpha_{ij}\;\geq \;0$, $\sum_{j}\alpha_{ij}=1$ encode the influence of neighbor *j* on target *i* based on their re*l*ative dynamics, and $v_{lj}=W_{l}^{V}h_{nbr,j}$ is the value vector.


$$\begin{array}{c} {head}_{i,l}=\sum_{j=1}^{N}\alpha_{ij}\;v_{lj} \end{array}$$ (7)

This yields a permutation-invariant social context that emphasizes the most influential neighbors. We explore three formulations for computing the attention weights $\alpha_{ij}$. In the standard neighborhood-based softmax formulation, the interaction weights are defined as Eq (8), where $s_{ij}$ denotes the similarity score between the target *i* and the neighbor *j*. Hence, $\alpha_{ij}$ measures how much the neighbor *j* influences the target of the attention head *l*. The similarity score $s_{ij}$ is then computed using one of several learnable functions (e.g., dot-product, additive, or distance-based attention) as expressed in Eq (9), where ‖ denotes vector concatenation and $d_{k}$is the dimension of each key/query vector). The attention weights are computed exclusive*l*y from the encoding position and related dynamics, allowing the model to identify behaviorally relevant neighbors without revisiting the raw coordinates or relying on hand-crafted features.


$$\begin{array}{c} \alpha_{ij}=\frac{\exp(s_{ij})}{\sum_{k=1}^{N}\exp(s_{ik})} \end{array}$$ (8)

$$\begin{array}{c} s_{lj}=\left\{ \begin{array}{cc} \frac{Q_{l}K_{l}^{\;⊤}}{\sqrt{d_{k}}}, & \text{scaled dot-product},\\ {}[6pt]w_{l}^{\;⊤}\tanh K_{l}, & \text{additive}(\alpha )-\text{attention},\\ {}[4pt]w_{l}^{\;⊤}[Q_{l}\;\|K_{l}], & \text{concatenation}, \end{array}\right. \end{array}$$ (9)

Moreover, while a single attention map can re-weight neighbors, a multi-head design probes complementary subspaces of trajectory features, e.g., one head emphasizing longitudinal gaps, another lateral incursions, and a third distant high-speed agents thereby enriching the interaction representation. Finally, the *M* head contexts are concatenated and linearly projected to form a single social context vector as expressed in Eq (10). Different heads discover complementary relations, e.g., short- vs. long-range motion features, lane-level vs. interaction-level context, which is crucial in trajectory prediction.


$$\begin{array}{c} H=\text{Proj}\;(h_{1}\|\cdots \|h_{m})\in {\mathbb{R}}^{H}. \end{array}$$ (10)

#### 2.2.3 Decoder.

The decoder produces the future trajectory $𝐘=\{({\hat{x}}_{t},{\hat{y}}_{t}){\}}_{t=1}^{T}$ conditioned on the encoder context $\left({𝐡}_{T},{𝐜}_{T}\right)$. At *t* = 0, the decoder is initialized with the encoder’s terminal states and *t*he last encoder input:


$$\begin{array}{c} {𝐡}_{0}={𝐡}_{T},\;{𝐜}_{0}={𝐜}_{T},\;{𝐱}_{0}={𝐲}_{T}. \end{array}$$

For t=1,...,T, the LSTM updates are expressed in Eq (11) and Eq (12). The output head maps the hidden state to a 2D position.


$$\begin{array}{c} {𝐡}_{t},{𝐜}_{t}=LSTM({𝐱}_{t-1},{𝐡}_{t-1},{𝐜}_{t-1}) \end{array}$$ (11)

$$\begin{array}{c} {\hat{𝐲}}_{t}={𝐖}_{\text{out}}{𝐡}_{t}+{𝐛}_{\text{out}},\;{\hat{𝐲}}_{t}\in {\mathbb{R}}^{2}. \end{array}$$ (12)

In an autoregressive setting, ${\hat{𝐲}}_{t}$ (or a teacher-forced target during training) is fed back as the next input ${𝐱}_{t}$, yielding a sequential rollout over the horizon.

## 3. Experimental evaluation

The proposed approach is evaluated using the publicly available highD dataset [50]. The details about the data set, data processing pipeline, training process, and the experimental setup can be found in our previous paper [49]. The data set is split such that 75% are used for training and 25% for testing. Each trajectory is segmented into an 8-second window, with the first 3 seconds serving as the observed input sequence and the remaining 5 seconds treated as the prediction horizon. Both the sequences are downsampled to 5 Hz. Two layers of LSTM are used during training with batch size of 32 and a learning rate of 10^−4^. The hidden state of LSTM encoder and decoder has a dimension of 128, and an output dimension of 2. RMSprop optimizer is used in combination with PyTorch framework and training is performed on an NVIDIA GeForce GTX 1080 GPU.

### 3.1 Evaluation metrics

The proposed model is evaluated using following metrics.

#### 3.1.1 Root Mean Square Error (RMSE).

RMSE measures the error between ground truth and predicted trajectories. In equation 13, $x_{ti}$ and $y_{ti}$ denote the predicted *x*,*y* coordinates of the *i*^th^ vehicle at time *t*, while $x_{ti}^{\prime }$ and $y_{ti}^{\prime }$ represent the corresponding actual coordinates. ${RMSE}^{t}$ denotes the RMSE at *t*ime *t*. Furthermore, $RMSE^{t}$ represents RMSE at time *t*.


$$\begin{array}{c} RMSE^{t}=\frac{\sqrt{\sum_{i=1}^{N}[(x_{ti}-x_{ti}^{\prime }{)}^{2})+(y_{ti}-y_{ti}^{\prime }{)}^{2})]}}{N} \end{array}$$ (13)

#### 3.1.2 Longitudinal error ($\varepsilon_{x}$).

Longitudinal error captures how far the predicted position is from the actual position in the direction of the vehicle motion. Given a trajectory along the x-axis, the longitudinal error is determined by the difference in predicted and actual x-positions.


$$\begin{array}{c} \varepsilon_{x}=||x_{pred}-x_{target}|| \end{array}$$ (14)

#### 3.1.3 Lateral error ($\varepsilon_{y}$).

Lateral error captures how far the predicted position is from the actual position in the direction perpendicular to the vehicle’s path. In Eq (15), $y_{pred}$ and $y_{target}$ the predicted and actual *y*-coordinates, respectively.


$$\begin{array}{c} \varepsilon_{y}=||y_{pred}-y_{target}|| \end{array}$$ (15)

### 3.2 Models compared

The performance of proposed model is compared with following state-of-the-art models.

1. AS-LSTM [28]: This model encodes vehicle interactions using a social-pooling layer.It also utilizes the self-attention mechanism to improve the weight distribution between inputs and outputs, which captures better positional effects.
2. Planning-informed prediction LSTM (PiP-LSTM) [51]: It applies planning information as an informed condition to produce multimodal trajectories.
3. NLS-LSTM [52]: A multi-modal prediction system based on an encoder-decoder architecture is utilized to provide a better insight into the variations in trajectories.
4. Multi-Head Attention Social Pooling (MHA-LSTM) [23]: The interaction modeling relies on a multi-head attention mechanism with four heads, though it works purely from vehicle position data, leaving other kinematic features.
5. Multi-Head Attention Social Pooling (MHA-LSTM(+f)) [23]: This model is an extension of the MHA-LSTM, where additional inputs like velocity, acceleration, and classes have been added along with three attention heads.
6. Sparse Graph Convolutional Network (SGCN) [53]: It models interactions by building a sparse directed spatial graph and learning information flow along its edges.
7. Scene LSTM Encoder-Decoder (S-LSTM) [54]: model is based on the social encoder-decoder model, which includes full connection pooling.
8. CS-LSTM [55]: It uses the temporal backbone of the target vehicle to predict its future trajectory by utilizing an LSTM. It ignores the effect of other vehicles around it in predicting trajectory.
9. LS-LSTM [56]: This model integrates an attention system within the traditional LSTM framework. It encodes both the target vehicle’s kinematic state and the behavioral patterns of surrounding vehicles across lanes, while dynamically weighing the relevance of each feature at every time step.
10. Dual Transformer [27]: This approach employs a dual-transformer architecture to simultaneously infer lane-change intent and predict vehicle trajectories. A multihead attention mechanism is utilized to characterize the interactions among target vehicle and surrounding vehicles.
11. Hybrid-LSTM [45]: This hybrid framework models how surrounding vehicles react to the intended trajectory of the ego vehicle using spatiotemporal attention. The attention mechanism adaptively weights past trajectories and inter-vehicle interactions, enabling the model to understand driving intent and predict motion more accurately.
12. Graph Recurrent Attentive Neural Process (GRANP) model [43]: This model leverages neural processes to directly quantify and visualize the uncertainty of the prediction. Its backbone couples a GAT with an LSTM and three stacked 1-D convolutional layers to model spatio-temporal dependencies efficiently.
13. GAT-TR-LSTM-LSTM [42]: This hybrid model fuses a GAT to model interactions on the road-agent graph and a transformer encoder to extract global temporal dependencies. An LSTM encoder captures local temporal patterns and the LSTM decoder generates future trajectories.

### 3.3 Ablation study

We performed an ablation study to identify key factors driving attention performance, examining the module along three complementary problems. The first problem is based on the number of attention heads. For this, models were trained with two to eight (2–8) attention heads to assess how multi-head diversity influences prediction accuracy. The second study is based on the type of attention method. For this, three attention methods are compared including α attention, scaled dot product, and concatenation-based attention. The third ablation study reports the performance parameters including inference time, number of parameters, number of FLOPs for different attention methods, and number of attention heads.

The findings of the first study are reported in next section. Using insufficient attention heads can not capture long range spatial and temporal dependencies appropriately. Prediction accuracy and the choice of attention head count must also be balanced against computational cost as more heads increase execution time and resource usage.

## 4. Results and discussion

For the first ablation study of the number of attention heads, we performed a series of experiments. For this purpose, our model is evaluated for prediction horizons 1-5s and number of attention heads varying from 2 to 8. We have provided RMSE, longitudinal and lateral errors in Table 2. It shows that increasing the number of attention heads from (h = 2) to (h = 3) yields consistent gains at all horizons with RMSE at 5s dropping from 0.87 to 0.68, corresponding reductions in longitudinal 1.07 to 0.82 and lateral errors 0.34 to 0.29. Using h = 4 is broadly comparable to h = 3, but performance degrades for h≥5 at 5s. RMSE increases to 0.67 with h = 6 and 0.69 with h = 8, while the longitudinal error increases to 0.81 and 0.85, respectively. These trends indicate a capacity fragmentation trade-off with fixed model width, larger ’h’ shrinks the per-head dimensionality $d_{k}$, diffuses attention and weakens long-horizon temporal coherence, while *h* = 5 provides the best balance of interaction modeling and stability over 1-5s. Considering the results, h = 5 is chosen for all subsequent experiments, as it offers the optimal trade-off in terms of interaction modeling and predictive robustness in both short- and long-term prediction perspectives.

**Table 2:** RMSE across 5s prediction horizon for varying numbers of attention heads in scaled dot-product attention on the highD dataset.

Prediction horizon (s)	Number of Attention Heads (h)					
	h = 2	h = 3	h = 4	h = 5	h = 6	h = 8
**RMSE (m)**						
1	0.11	0.08	0.09	0.09	0.09	0.09
2	0.25	0.18	0.19	0.18	0.18	0.19
3	0.45	0.30	0.32	0.29	0.31	0.31
4	0.66	0.47	0.49	0.44	0.47	0.47
5	0.87	0.68	0.67	0.61	0.67	0.69
**Longitudinal Error (m)**						
1	0.14	0.11	0.12	0.11	0.11	0.11
2	0.32	0.22	0.24	0.23	0.23	0.23
3	0.56	0.37	0.39	0.36	0.37	0.39
4	0.82	0.57	0.60	0.53	0.57	0.58
5	1.07	0.82	0.81	0.72	0.81	0.85
**Lateral Error (m)**						
1	0.04	0.03	0.03	0.04	0.04	0.04
2	0.10	0.08	0.08	0.08	0.08	0.08
3	0.17	0.14	0.14	0.14	0.13	0.13
4	0.26	0.21	0.21	0.21	0.21	0.20
5	0.34	0.29	0.29	0.28	0.28	0.27

The second ablation study is carried out on the type of attention method used. For this purpose, our model is evaluated for α-attention, scaled dot-product, and concatenation attention over the 1-5s prediction horizon. Table 3 shows that the scaled dot product achieves the lowest RMSE (0.09 to 0.29) for horizons 1-3s. However, at the 4s and 5s horizon, concatenation yields the lowest RMSEs of 0.39 and 0.35, respectively. *alpha* attention remains at highest RMSE at all horizons except 1s. The reason is that it does not consider encoding vectors of the target and relies on encoding vectors of the surrounding vehicles only. These results indicate that the concatenation yields better logit calibration and more stable long-horizon behavior, and the scaled-dot product performs well in short horizons.

**Table 3:** Performance over a 5s prediction horizon for different attention mechanisms.

Prediction	α-Attention			Scaled Dot-Product			Concatenation		
Horizon (s)	RMSE	Long.	Lat.	RMSE	Long.	Lat.	RMSE	Long.	Lat.
1	0.09	0.12	0.03	0.09	0.11	0.04	0.10	0.13	0.05
2	0.25	0.31	0.09	0.18	0.23	0.08	0.23	0.28	0.11
3	0.45	0.56	0.17	0.29	0.36	0.14	0.34	0.41	0.17
4	0.66	0.82	0.26	0.44	0.53	0.21	0.39	0.45	0.21
5	0.89	0.98	0.35	0.61	0.72	0.28	0.35	0.37	0.23

**Fig 3:**
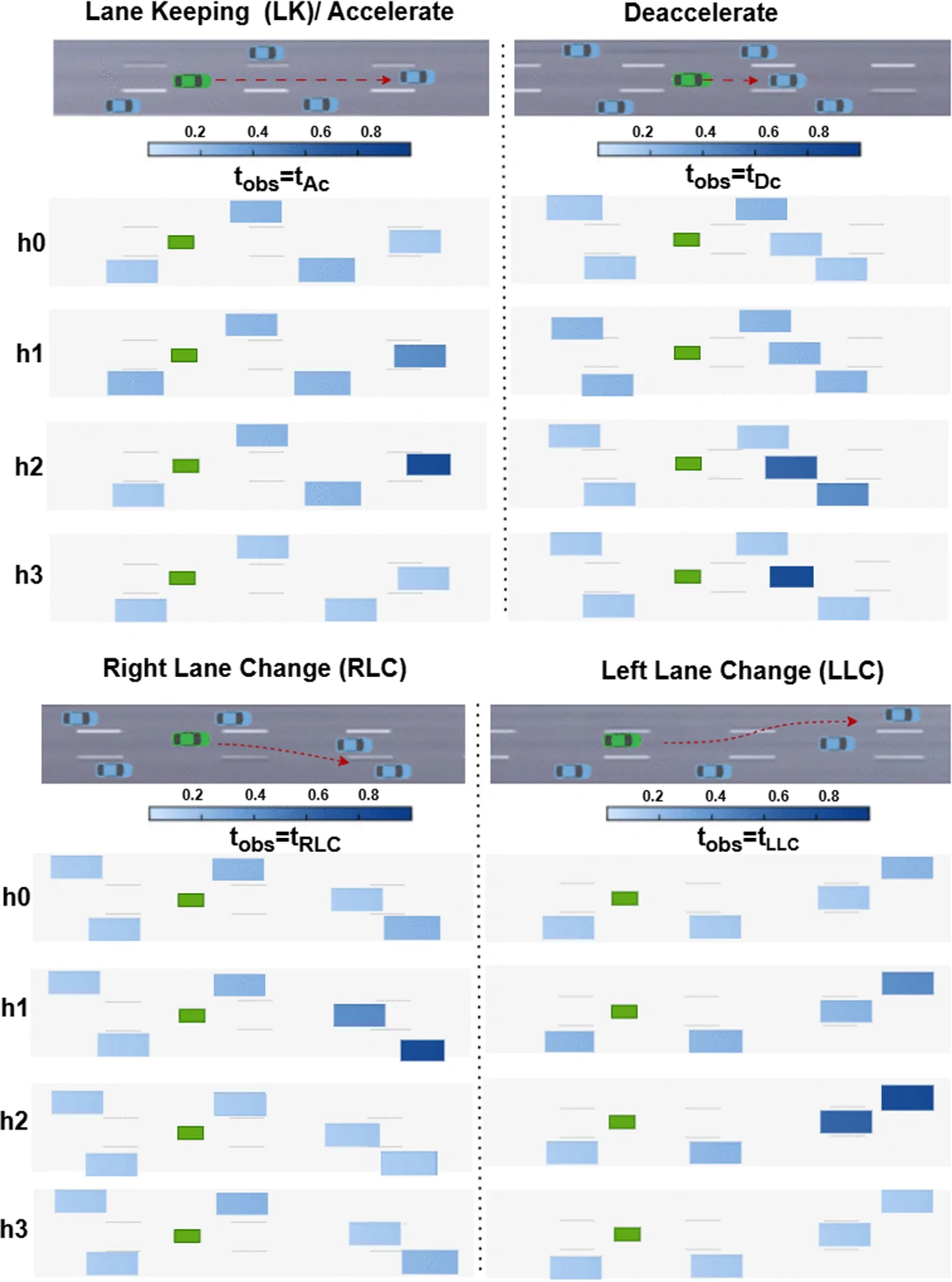
Attention maps for four different driving scenarios.

For qualitative analysis of attention heads, Fig 3 shows four heads attention maps for lane keeping/accelerate, de-accelerate, right and left lane-change driving scenarios. The four attention heads are represented as *h*_0_....*h*_4_. The target vehicle is represented with green, and its neighbors are shown in blue color. The color of neighbors indicates their attention weight; it is darker when they have more weight. We can remark that the vehicles in front of target vehicle are assigned a higher attention weight compared to the vehicles following it. In each scenario, there is one head that assigns equal weight to all surrounding vehicles. In lane keeping (*h*_3_), de-accelerate (*h*_1_), right lane change (*h*_2_) and left lane change (*h*_3_). Similarly, there is one attention head that assigns the most distinctive weights to all neighbors in each scenario. In lane keeping (*h*_2_), de-accelerate (*h*_2_), right lane change (*h*_1_) and left lane change (*h*_2_). We can also notice that in the left lane change scenario (*h*_2_), the vehicle at the longest distance is assigned the most weight and the closest neighbors are assigned less weight. The closest neighbors do not have the greatest influence in predicting the trajectory of the vehicle.

**Fig 4:**
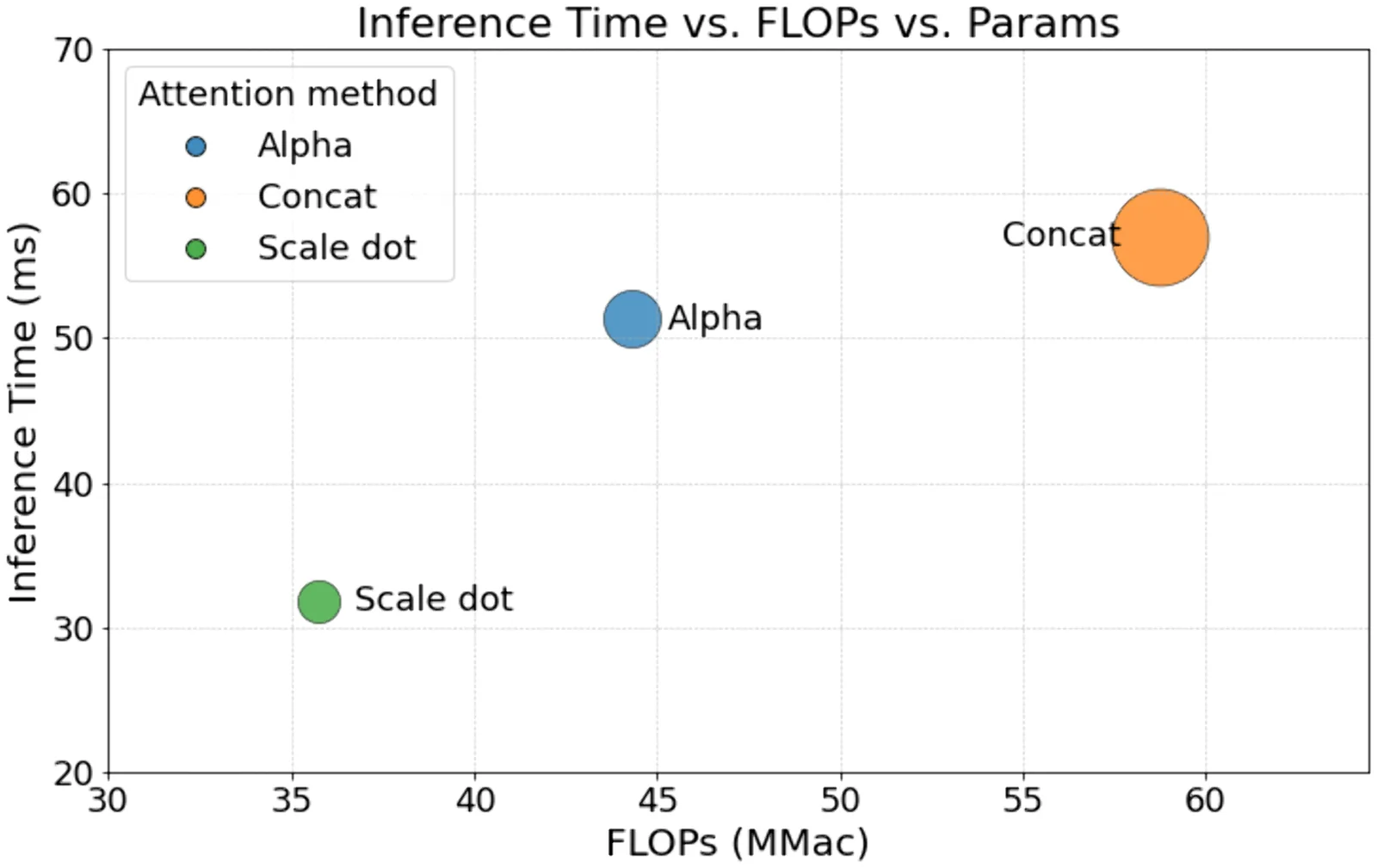
Comparison across different attention methods.

**Fig 5:**
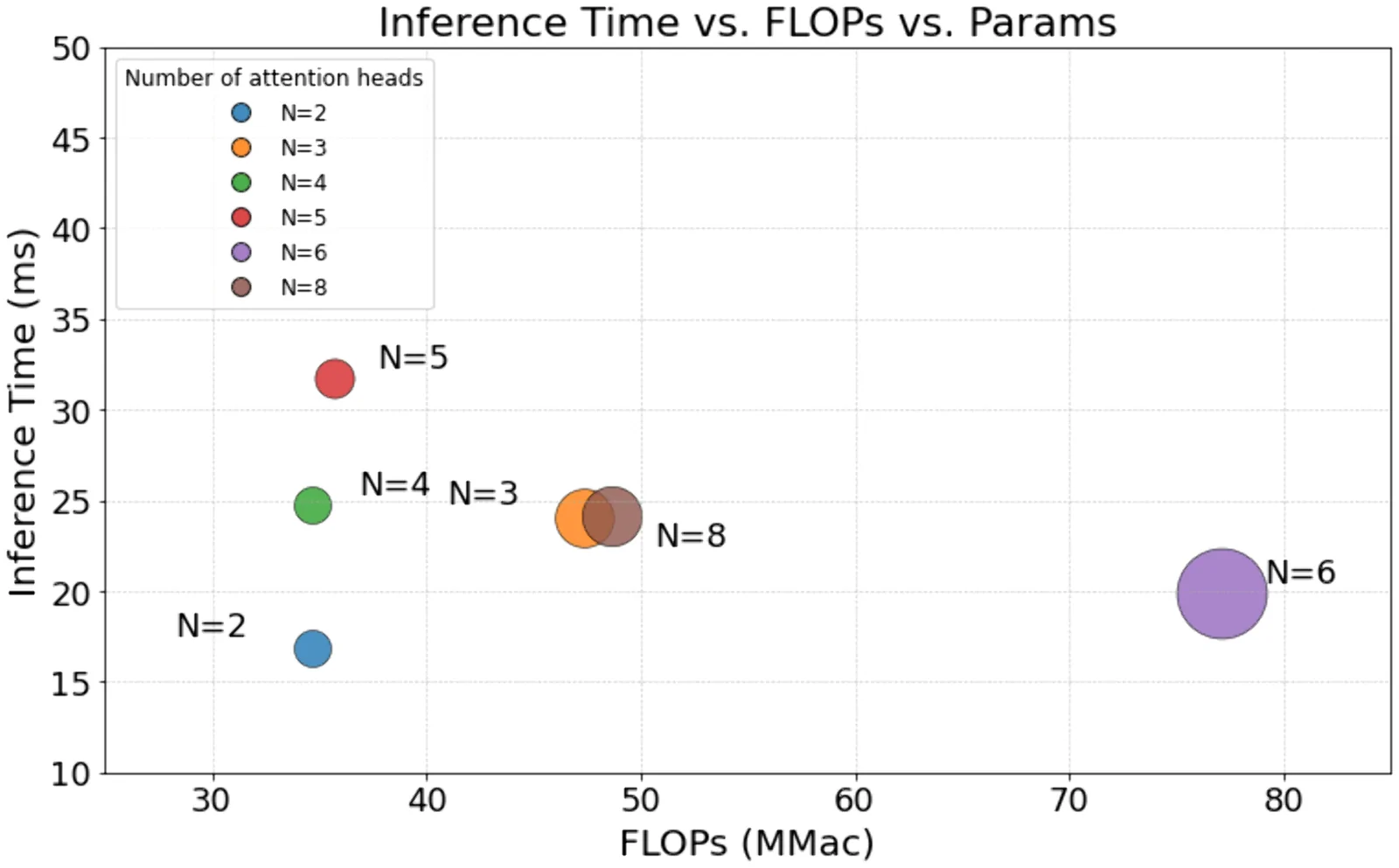
Comparison across different numbers of attention heads.

The third ablation study reports the inference time, the number of parameters, the number of FLOPs for different attention methods, and the number of attention heads. Fig 4 shows the performance of our model with different attention methods. The bubble sizes represent the parameters of the proposed model. The scaled dot attention (green bubble) exhibits lower FLOPs and latency because its scores include batched dot products and a softmax. Alpha attention (blue bubble) involves a nonlinearity and a learned vector which increases parameter count and latency moderately. Concat attention (orange bubble) doubles the score input dimension due to query and key vector stack, resulting in higher FLOPs, latency, and a larger parameter bubble. Overall, scaled dot-product can be a good choice if compute or latency-bounded as it appears fast and cheap. We can choose additive or concatenation attention only when they provide higher accuracy to justify the extra cost. Fig 5 presents performance of our model using different number of attention heads. During these experiments, the scale-dot attention method is used. Practically, a moderate head count of four to five is a favorable latency and FLOPs trade-off. Using more than five attention heads should be considered only when they deliver measurable accuracy that justify the added runtime and memory budget.

Table 4 reports the statistical validation of proposed NL-MHA-LSTM model across five independent runs with different random seeds. RMSE increases from 0.093m at 1s to 0.675m at 5s, reflecting growing prediction uncertainty over longer horizons. The narrow confidence intervals across all metrics confirm that the model converges consistently and its performance is reproducible rather than dependent on a single favorable initialization. Lateral errors remain consistently lower than longitudinal errors across all horizons, which is expected given the structured lane-keeping behavior characteristic of highway driving.

**Table 4:** Statistical validation of the proposed NL-MHA-LSTM model.

Metric		Mean	Std	95% CI [Low, High]
RMSE	1s	0.0932	±0.0024	[0.0872, 0.0992]
	2s	0.1919	±0.0109	[0.1647, 0.2190]
	3s	0.3183	±0.0232	[0.2607, 0.3759]
	4s	0.4884	±0.0399	[0.3892, 0.5875]
	5s	0.6749	±0.0611	[0.5230, 0.8268]
ADE		0.3009	±0.0221	[0.2461, 0.3558]
FDE		0.6749	±0.0611	[0.5230, 0.8268]
Longitudinal Error	1s	0.1160	±0.0021	[0.1108, 0.1212]
	2s	0.2341	±0.0135	[0.2006, 0.2677]
	3s	0.3809	±0.0298	[0.3069, 0.4550]
	4s	0.5818	±0.0527	[0.4509, 0.7128]
	5s	0.7987	±0.0820	[0.5948, 1.0025]
Lateral Error	1s	0.0416	±0.0024	[0.0357, 0.0475]
	2s	0.0890	±0.0044	[0.0781, 0.0999]
	3s	0.1543	±0.0077	[0.1351, 0.1736]
	4s	0.2355	±0.0111	[0.2079, 0.2630]
	5s	0.3179	±0.0152	[0.2800, 0.3557]

### 4.1 Performance evaluation of NL-MHA-LSTM against existing attention-based models

Table 5 benchmarks NL-MHA-LSTM against several attention-based models on the HighD dataset, including AS-LSTM [28], PiP-LSTM [51], NLS-LSTM [52], MHA-LSTM [23], MHA-LSTM(+f) [23], SGCN [53], S-LSTM [54], CS-LSTM [55], LS-LSTM [56], Dual Transformer [27], Hybrid-LSTM [45], GRANP [43], and GAT-TR-LSTM-LSTM [42]. AS-LSTM combines social pooling with a self-attention mechanism built on scaled dot-product attention, enabling it to learn cooperative driving behaviors and inter-vehicle interactions effectively. This design places it among the top performers at shorter horizons (1-4s). The performance of AS-LSTM becomes extremely poor for longer prediction horizons (5s: 0.93), however, due to the limitation of its self-attention mechanism for preserving temporal dependencies over extended prediction horizons.

**Table 5:** Comparison of RMSE(meters) among various attention-based models using HighD dataset.

Prediction Horizon (s)	AS- LSTM [28]	PiP [51]	NLS- LSTM [52]	MHA-LSTM LSTM [23]	MHA-LSTM (+f) [23]	SGCN [53]	S-LSTM [54]
1	0.06	0.16	0.20	0.19	0.06	0.15	0.22
2	0.08	0.47	0.57	0.55	0.09	0.38	0.62
3	0.19	0.94	1.14	1.10	0.24	0.72	1.27
4	0.43	1.58	1.90	1.84	0.59	1.16	2.15
5	0.93	2.36	2.91	2.78	1.18	1.71	3.41
**Prediction** **Horizon (s)**	**CS-** **LSTM** [55]	**LS-** **LSTM** [56]	**Dual** **Transformer** [27]	**Hybrid-LSTM** [45]	**GRANP** [43]	**GAT-TR-** **LSTM** [42]	**NL-MHA** **LSTM**
1	0.22	0.30	0.41	0.16	0.41	0.15	**0.04**
2	0.61	0.38	0.79	0.47	0.44	0.37	**0.07**
3	1.24	0.45	1.11	0.94	0.70	0.66	**0.18**
4	2.10	0.60	1.40	1.58	0.88	–	**0.33**
5	3.27	0.88	–	2.38	1.34	–	**0.41**

The MHA-LSTM(+f) model incorporates additional kinematic and class-type features with multi-head attention mechanism, including velocity, acceleration, and vehicle class, using scaled dot-product attention. This enriched feature set translates into strong short-range predictive performance, achieving an RMSE of 0.06m at 1s, placing it second overall at that horizon. Nevertheless, even these models struggle with long-range predictions due to the problems associated with memory capacity and prediction error accumulation. Methods that rely mainly on the target vehicle’s history S-LSTM, CS-LSTM, PiP-LSTM and Hybrid-LSTM exhibit markedly higher RMSE as 3.41, 3.27, 2.36 and 2.38 at 5s, underscoring the limitations of models without explicit interaction modeling. In contrast, our NL-MHA-LSTM achieves 0.41 at 5s, representing a 53.4% reduction in comparison to the next-best with 0.88 RMSE (LS-LSTM). The graph and transformer variants such as SGCN and Dual Transformer are competitive at short horizons, these approaches degrade more steeply as the prediction window extends. Among these models, NL-MHA-LSTM yields the most stable long-range prediction performance (5s: 0.41). These results are reported using the best-performing checkpoint during training process.

**Fig 6:**
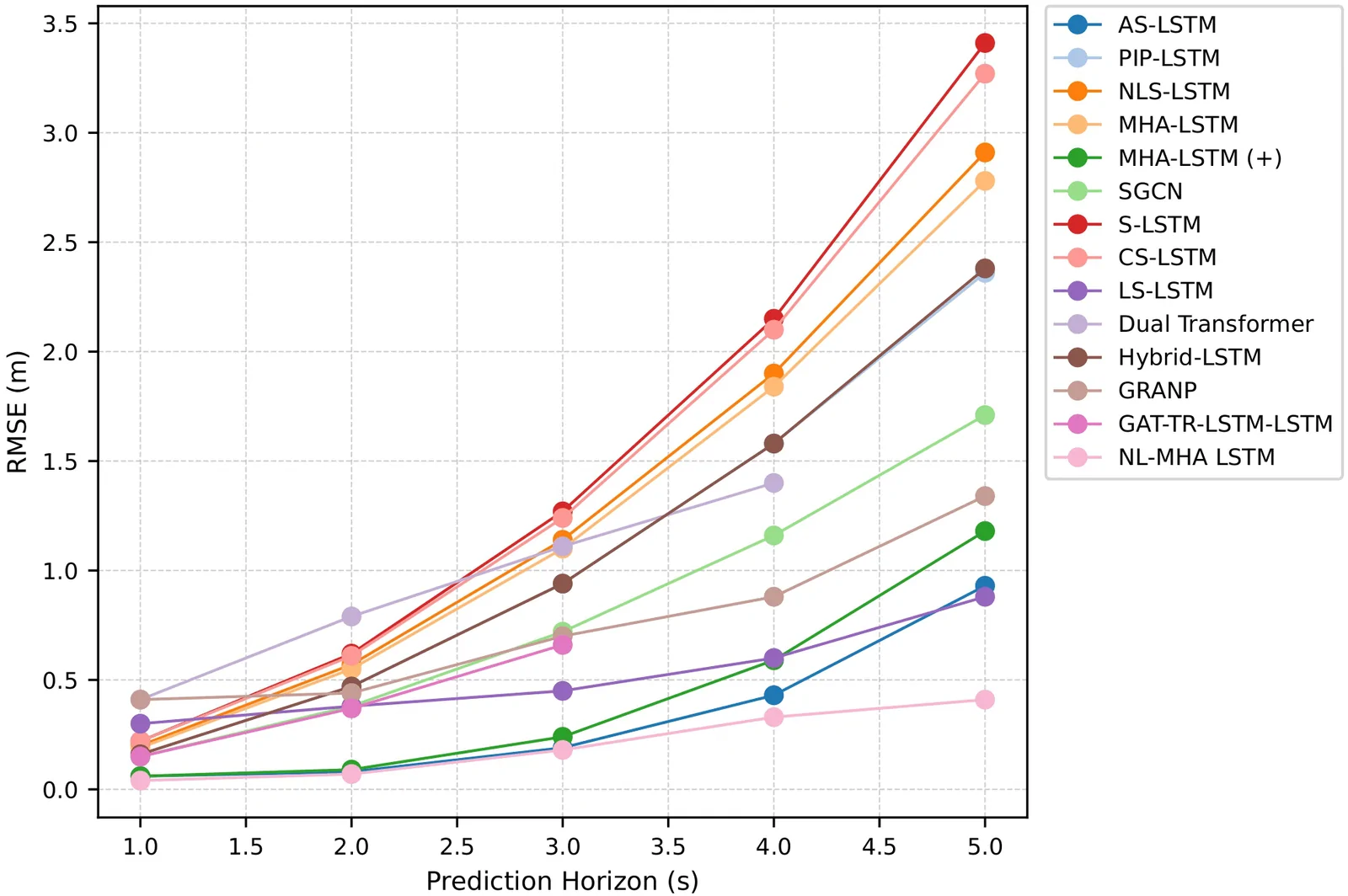
Distribution of RMSE over prediction horizons for different models.

Fig 6 illustrates how the RMSE of each model evolves across prediction horizons ranging from 1-5s. Our model shows better performance for longer horizons providing the best results on all horizons. It proves the significance of developing spatiotemporal modeling techniques and using proper features for making accurate predictions on longer horizons. The experimental results emphasize the weaknesses of the current approaches in dealing with long-term predictions. Our proposed model is stable on all horizons and demonstrates the best performance on longer horizons. The reason is improved feature representation, optimized training and attention mechanism.

### 4.2 Scenario-specific comparison

The evaluation compares proposed NL-MHA-LSTM against a set of established attention-based models for vehicle trajectory prediction. NST-LSTM [49] is not included in above comparison (Table 5) because it uses a fixed uniform pooling rather than an attention mechanism. A dedicated scenario-specific analysis is performed for comparison between NST-LSTM and NL-MHA-LSTM.

To better understand where the performance gains actually come from in both models, Table 6 outlines lane-following and lane change scenarios extracted from the HighD dataset. Out of 110,516 trajectories, 24,617 fall into the lane-following category; where interactions between vehicles are sparse but they carry significant weight. The remaining 85,899 trajectories involve lane-change scenarios, where interactions are dense. Table 7 shows the RMSE for both scenarios. In lane-following scenarios, NL-MHA-LSTM shows 1.50m RMSE and NST-LSTM reduces this by approximately 50%, achieving 0.75m. In lane change scenarios, the trend reverses: NST-LSTM achieves an RMSE of 0.52m compared to 0.70m for NL-MHA-LSTM. NL-MHA-LSTM demonstrates better performance in lane-following scenarios, where the ability to selectively assign attention weights to the most relevant neighboring vehicles is critical, while NST-LSTM performs better in lane-change scenarios where uniform pooling is sufficient. The overall RMSE favors NST-LSTM simply because HighD is dominated by dense lane-change scenarios.

**Table 6:** Scenarios in HighD dataset.

Scenario	Total Trajectories
Total trajectories	110,516
Lane-following	24,617
Cut-in / lane-change	85,899

**Table 7:** RMSE comparison across different traffic scenarios.

Model	Lane-Following RMSE (5s)	Lane-Change RMSE (5s)
NST-LSTM [49]	1.50	0.52
NL-MHA-LSTM	0.75	0.70

Table 8 compares the computational complexity of NST-LSTM and the three NL-MHA-LSTM attention variants. NST-LSTM is the most lightweight, with an inference time of 8.015ms and 493.83K parameters, due to the absence of an attention module. The NL-MHA-LSTM variants are already discussed in section 4. The higher computational cost of NL-MHA-LSTM over NST-LSTM is a direct consequence of the attention mechanism, which enables learned neighbor weighting and improved model explainability.

**Table 8:** Computational complexity comparison of NST-LSTM and NL-MHA-LSTM.

Model	Inference Time (ms)	FLOPs ($\times 10^{6}$)	Parameters (K)
NST-LSTM [49]	8.015	50.77	493.83
NL-MHA-LSTM (Alpha Attention)	51.30	88.56	863.46
NL-MHA-LSTM (Scale Dot Attention)	31.77	71.46	765.73
NL-MHA-LSTM (Concat Attention)	56.97	117.42	1,140.00

### 4.3 Limitations

The proposed NL-MHA-LSTM has several limitations. The model is trained and evaluated exclusively on the highway dataset, highD. This limits the scope of applicability of NL-MHA-LSTM in more complicated or unstructured scenarios like urban intersections, roundabouts and high occlusion traffic. Extending evaluation to other datasets is not straight-forward, most alternative datasets require substantial custom feature engineering. The model predicts the future position of the target vehicle only, without predicting velocity or acceleration, which limits its direct utility for motion-planning modules that require full kinematic state estimates rather than position alone. In addition, the decoder produces a single deterministic trajectory rather than a distribution over plausible future. The real driving behavior is inherently multi-modal; a vehicle approaching a gap in traffic may accelerate through it, cautiously merge, or yield entirely depending on contextual patterns that are difficult to capture from position alone.

## 5. Conclusion and future work

This paper introduces an attention-based multi-head spatiotemporal attention model that captures the interaction among vehicles. The proposed model captures long-range spatiotemporal interactions between the vehicles without any constraints that limit conventional neighborhood-based approaches. The high-order interactions are processed by deep LSTM encoder. We subsequently use the attention mechanism to weight encoded interaction features by relative position and motion. It emphasizes key vehicles and delivers strong position-aware features. The experimental evaluations show that the approach suggested performs much better than existing techniques concerning RMSE, longitudinal error, and lateral error. Besides, the ablation study conducted for attention types, head numbers, and performance parameters has shown that parameter selection is crucial for an attention mechanism. The evaluation findings suggest that combining non-local spatiotemporal interaction modeling with a multi-head attention mechanism and a deep LSTM architecture collectively drives the prediction accuracy gains observed in highway driving scenarios.

Future work will evaluate this framework on unstructured traffic environments, such as intersections and roundabouts with the goal of improving model’s generalization and prediction capability. For generalization, the proposed model will be tested on other publicly available datasets like INTERACTION [57], nuScenes [58], and V2X-Sim [59,60] including more diverse traffic scenarios. For a richer feature space, kinematics (speed, acceleration) and semantics (vehicle type) will be integrated in the model. In addition, future work will include generating a distribution of possible future trajectories instead of one deterministic output to reflect the inherent multimodality of real-world driving.
